# Calibration of Smartphone-Based Weather Measurements Using Pairwise Gossip

**DOI:** 10.1155/2015/494687

**Published:** 2015-09-02

**Authors:** Jane Louie Fresco Zamora, Shigeru Kashihara, Suguru Yamaguchi

**Affiliations:** Graduate School of Information Science, Nara Institute of Science and Technology, 8916-5 Takayama, Ikoma, Nara 630-0192, Japan

## Abstract

Accurate and reliable daily global weather reports are necessary for weather forecasting and climate analysis. However, the availability of these reports continues to decline due to the lack of economic support and policies in maintaining ground weather measurement systems from where these reports are obtained. Thus, to mitigate data scarcity, it is required to utilize weather information from existing sensors and built-in smartphone sensors. However, as smartphone usage often varies according to human activity, it is difficult to obtain accurate measurement data. In this paper, we present a heuristic-based pairwise gossip algorithm that will calibrate smartphone-based pressure sensors with respect to fixed weather stations as our referential ground truth. Based on actual measurements, we have verified that smartphone-based readings are unstable when observed during movement. Using our calibration algorithm on actual smartphone-based pressure readings, the updated values were significantly closer to the ground truth values.

## 1. Introduction

The Intergovernmental Panel on Climate Change (IPCC) reported on extreme changes in climate beginning in the 1950s. Moreover, significant increases in the frequency of heavy precipitation and its intensity were forecasted to occur in this century [1]. With the decline in the number and quality of global weather daily reports [2], this becomes a problem when dealing with rain-related disasters. Examples of these disasters are very strong winds that typically occur before and during the rain, as well as flash flooding and landslides that usually occur during or after the rain has happened. This is especially true for Asia with reported total deaths of 1,005,608 (in thousands) caused by flooding and landslides [3].

To observe rain events or the atmospheric condition in general, meteorologists use weather instruments to measure the temperature, wind, pressure, and humidity of the environment [4]. This observation system is a combination of in situ weather devices, radars, and geostationary satellites monitoring the atmosphere. Recently, the national meteorological services (NMS) employed automatic weather stations for ground observations and weather radars with increased spatial and temporal resolutions. For example, surface observations by the Japan Meteorological Agency are provided by their Automated Meteorological Data Acquisition System (AMeDAS), which is composed of 1,300 automatic weather stations [5]. These weather stations are sparsely distributed all over the country on an average of 17 km apart. Meanwhile, the USA obtains surface weather information from their Automated Surface Observing System (ASOS), which also reports basic weather elements similar to AMeDAS [6]. Furthermore, the development of phase-array radars is ongoing and a few X-band radars have already been installed in selected prefectures in Japan. These instruments have resolutions of several 100 meters in area and can provide data within the span of seconds to a few minutes. However, the wide use of these sophisticated instruments, especially in developing countries, has yet to materialize.

Additionally, there are emerging private companies that provide weather information as a service. To name a few, they are Weathernews, Inc. (WNI) [7] and the Japan Weather Association (JWA) [8] based in Japan, Weather Underground [9] and Accuweather [10] in the US, and WeatherOnline [11] and MeteoGroup [12] in Europe. A majority of these companies cater to clients who need customized weather information from sea to land. The resources of these institutions are diverse and wide, which enables them to deliver accurate and prompt information that, however, comes with a cost. WNI, for instance, provides notifications on warnings for localized heavy rains only to individuals who are registered as clients. This particular feature is only available when an individual pays for the required monetary dues. In general, companies and businesses avail of these weather information services for the continuous operation of their enterprise. Thus, only individuals who are willing to pay for the monthly service dues and add-on payments would typically avail of this service.

Considering the need for high resolution surface weather data in forecasting extreme rain-related events, we need to examine low-cost and low-maintenance solutions. The additional installation of high-end weather instruments that are typically used by NMSs would normally depend on the national budget. Also, aside from purchasing these peices of equipment, the government may also need additional labor to maintain them. As for private weather services, it may not be practical for some individuals especially in developing countries to pay a monthly fee if they only need updates on particular days. They may opt for the free weather service by the government but the information would typically be insufficient and sometimes inaccurate. As a solution, researchers find alternatives in sensor network technologies to observe ground surface weather. SensorScope [13], for example, is a weather station network composed of assembled commercial environmental sensors that are robust and can be deployed in remote areas with low maintenance. Another approach to surface weather observation is by using rain-induced signal attenuation in microwave links to observe and characterize rain events as in [2, 14]. Even with these developments, however, we may need more observation points of high spatial and temporal resolutions especially for understanding heavy precipitation that typically occurs at scales of subkilometer.

A prior work [15] on using distributed devices for surface weather observation suggested the need for a calibration method to effectively and reliably aggregate weather information. As one of the solutions to address the problem of collaborating different types of devices, narrowing the focus to smartphone-based measurements can be a fundamental approach. Thus, analyzing measurements from embedded smartphone sensors would be a better way of obtaining high density synoptic weather information considering that many people currently use smartphones. However, one problem is that a smartphone is not necessarily a fixed sensor and is typically, for instance, kept inside trouser or shirt pockets and bags or held in-hand for sending messages or calling. This could obviously affect the measurement quality if we aim for a continuous long-term data gathering. For example, in most cases, portable environmental sensors like the ones embedded in the smartphones are helpful when the user would like to find out current weather conditions. It is on this instance that the user holds out the device and obtains the instantaneous measurements. Nonetheless, we would like to record the environmental conditions even when the user does not deliberately perform the measurements. This is so because we try to take advantage of the possibly large amount of data that we could provide to general forecasting services especially for predicting extreme rain-related events. Thus, we propose to utilize smartphones, smartphone-based sensors, and other existing commercial weather sensors to provide additional and high-dense information of surface ground weather to support general forecasting services.

Similar to how weather instruments are calibrated and maintained, however, smartphone-based data should undergo similar data correction processes to yield reliable synoptic weather data. Therefore, in this paper, we investigate how to correct or adjust smartphone-based environmental data even when the device is normally used as a smartphone. Using commercial weather instruments and built-in smartphone sensors, we adjust the device-based measurement with a heuristic-based pairwise gossip algorithm. In our general setup, fixed sensors like the commercial weather stations are assumed to produce proper information and therefore the majority of the adjustments are to be performed on the smartphone-based data. To do so, we consider the basic context-based information such as acceleration to observe user activity and adjust the measured information accordingly.

Quantitative estimation of the current environmental conditions, such as temperature, humidity, and pressure, allows general forecasting services to have an outlook of the weather in the next few minutes partly based on ground surface information. If the estimate is far from actual conditions, this could remarkably affect the calibration with other weather instruments and eventually the forecasting model outputs. Therefore, if we could accordingly adjust the values from the source in reference to a relative ground truth, then we may be able to mitigate forecasting errors from the lowest level of computation. The contribution of this paper is the formulation of a heuristic-based pairwise gossip algorithm that will adjust pressure values as measured by the embedded sensors in the smartphone based on a normal usage. Adjusting the smartphone-based value when the user is stationary or moving requires the reference measurements of a weather station as our established ground truth. In this way, it does not require complex formulations to easily calibrate the pressure sensor on the smartphone.

The rest of the paper is divided into detailed discussions as follows.  Section 2 provides a brief background on mobile phone sensing and sensor network calibration.  Section 3 discusses our experimentation with embedded smartphone pressure sensors.  Section 4 presents the heuristic-based pairwise gossip algorithm that will adjust environmental data accordingly with a fixed weather station. Lastly, Section 5 summarizes the paper and discusses future work.

## 2. Related Work

When mobile phones with position, motion, and environmental sensors began to be manufactured, it gradually became a recognized device for sensor networks. Examples of such embedded sensors are the accelerometer, gyroscope, light meter, proximity sensor, magnetometer, pressure, temperature, and humidity sensors [19]. Lane et al. [20] emphasized that the conception of mobile phone sensing (MPS) research field is due to a variety of feasible applications using mobile phone sensors. These applications range between social network services, environmental monitoring, and personal health improvement. A typical example of an MPS application would be user context recognition systems [21] that aim to deliver better services based on user behaviour, for instance. Context includes user activities and interactions with other users or with the environment that is based on the MPS data. More often, to get a general view of a group of users, the data has be to be sourced from a multiple group of users having similar qualifying criteria. These criteria are based on categorization of MPS data by statistical methods like mean, median, variance, and so forth, to analyze patterns in the data and group-like patterns accordingly. In addition to context recognition, which frequently makes use of position or motion sensor data, environmental application of MPS as listed in [22] also includes utilizing microphones and cameras to provide audio and image samples of the environment, respectively. Another example of environmental MPS is discussed in [23] where it makes use of the temperature sensor to estimate urban air temperatures. With recent mobile phone models like those in Table 1, it is now possible to observe pressure, ambient temperature, and relative humidity for meteorological applications.

However, “crowdsourced” environmental data is largely affected most especially by how individuals are using their devices when data is taken. Several surveys like [24–26], for instance, reveal that individuals would typically keep the devices inside their shirt or trousers pockets or inside shoulder bags or backpacks. Although these survey results were mainly used for activity recognition, we expect that measurements performed in such instances may offset the ideal measured value and notably affect the accuracy and reliability of environmental analysis. Thus, in [27], it has been emphasized that calibration is important in sensor networks to avoid unreliable measurements. This is typical for environmental sensors which weather forecasts rely on and significant for crowdsourced data affected by several human factors. Furthermore, calibration allows for the identification of errors in the system that may be attributed to offset faults, gain faults, and drift faults. While calibration is often a difficult task, it can be typically implemented in sensor networks before they are deployed or while they are on deployment. An example of calibration that is performed on sensors in situ is the work on target detection using low-cost sensors as in [28]. The study proposed a calibration algorithm based on feedback control theory and a combination of data fusion and Bayesian detection models to properly identify a target exposed under the sensors. Results from small-scale testbed and simulation using real vehicle detection data have proven that target detection using their algorithm achieved optimal performance. Meanwhile, another approach to calibrating sensor networks is based on a gossip protocol [29]. The goal was to estimate a signal signature based on the collective sensor node values while calibrating the values at the same time. Distributed processing techniques were applied to uncalibrated sensors in the network to correct them and determine the signal pattern. Based on their system model and the gossip-based distributed algorithm, the distributed signature learning and node calibration (D-SLANC) algorithm was derived. This algorithm enables local calibration among sensor nodes and addresses the global estimation problem.

Similarly in this paper, we would like to address faults with the embedded smartphone sensors with high-end commercial weather stations as our relative ground truth. In this way, we may be able to utilize the smartphones as a network of weather instruments that can provide general forecasting services with sufficient synoptic ground weather information for their forecasts. Thus, using analytical techniques of context recognition like in [30, 31] and gossip-based concepts, we would like to investigate the effect of placing the device inside a shoulder bag with some user activity in our aim to correct embedded smartphone sensor measurement at the device level.

## 3. Investigation of Smartphone Sensor Data as Affected by User Activity

Based on the survey results of mobile phone usage provided in [25], 35% of the respondents put their devices inside a bag. This is greater than both the 30% of respondents who put it inside their trouser pocket and 13% who put it inside the chest pocket. Therefore, considering these statistics, we chose to observe first the effect of placing the device inside a bag using two experiments. Using several models of Samsung smartphones listed in Table 1, we investigate on the pressure data having the sensor common to all devices in the list, while the device is measuring from inside a shoulder bag. Surface pressure is an indication of the changes in atmospheric forces that is helpful for meteorologists to predict what kind of weather we will be experiencing. For now, we simply focus on the pressure readings for the weather measurement since the environmental sensor is most common to some smartphone models which are currently being manufactured. Each device is then installed with an Android application that we developed, which logs the available sensor data for every second. The application, in general, samples the instantaneous measurement of the embedded smartphone sensors at every second and then continuously logs these values as a CSV file in the internal device storage.

We used two units of the Samsung S3 models and one unit each of the Samsung Galaxy Nexus and S4 models, subjecting four devices overall in both experimental setups. Both experimental setups used the same shoulder bag by the same user to perform the measurements. Motion sensors, such as the gyroscope, accelerometer, and magnetometer, were observed in three dimensions subjective to the device orientation. Meanwhile, environmental sensors like light, proximity, pressure, temperature, and humidity were recorded as is. In both experiments, we refer our readings to high-end commercial weather stations, such as the Vaisala WXT520 [32] to provide us with measurements for our estimation reference. It is important to note that we are using several devices, each of which has a particular margin of error. As for the Vaisala, it has an acceptable error of ±0.5 hPa considering that it has been calibrated according to standard. The pressure sensor in the smartphones has a maximum absolute error of 4 hPa by the specifications according to [33]. Considering these errors, we cannot directly compare the accuracy of smartphone devices to the Vaisala since each instrument was developed for different purposes. However, we find that the pressure sensors in the smartphones can be proven to be useful if calibrated accordingly and if there is potentially enough data. By sufficient data, this could mean having at least one available smartphone device in a 100-meter unit area.

**Figure 1:**
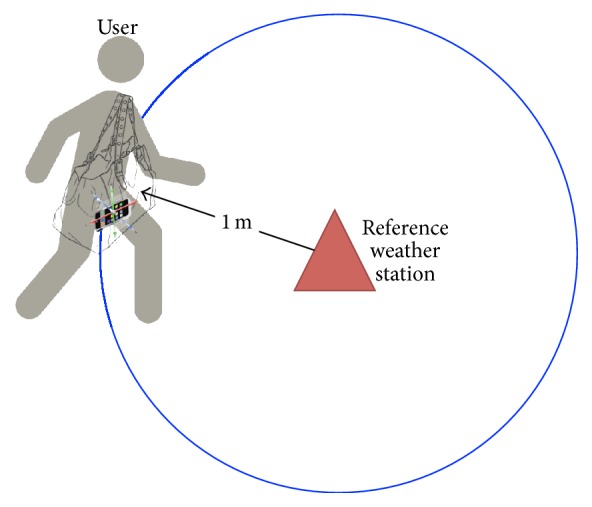
Setup of the stationary user experiment.

Generally in our experiments, we measure within 10-meter distance as an example of similar measurement situations. Pressure measurements do not significantly change in several hundred meters horizontally but the change in terrain will. In particular with smartphone devices as point sensors, the reported pressure may not be similar when measured in an elevated area as opposed to flat land within several hundred meters. As for the first experiment, it was conducted to investigate the precision of the barometric readings by smartphones compared with that of the weather station. A bigger picture of this scenario is when a user is idling nearby a fixed weather station and with the device measuring on the background. In the setup shown in Figure 1, the user was required to stand one meter from the reference weather station while carrying the shoulder bag with the devices inside it. A meter away from the weather station minimizes the influence on the instrument. The measurements were performed for three separate afternoons while sampling sensor data for 10 minutes in each event. Studies on context recognition would typically sample for one to a few minutes to get enough dataset. For similar experiments to ours, the duration may vary depending on the desired sample size. In our case, we decided on 10 minutes to get enough samples of both environmental and motion sensors. Also, the reference weather station measures every minute and 10 samples are sufficient to describe the surface pressure. Before determining how precise the smartphone-based pressure readings with that of Vaisala, we first preprocessed the data. As the observations were logged in seconds, we wanted to match the per minute resolution of the weather stations. To do so, we sampled an overlapping window on the same minute (60 units) and determined the median. We use these values of median per minute and implemented them in the following uncertainty range equation *α* as in

(1) $$\begin{array}{c} \alpha =\left(\bar{X\left(t\right)}-x_{\min}\left(t\right)\right)+\left(x_{\max}\left(t\right)\right)-\bar{X\left(t\right)}, \end{array}$$

where $\bar{X(t)}$ is equal to ∑_*i*=1_^*n*^ *x*_*i*_(*t*) + *x*_ref_(*t*) divided by *N* for *n* = 4 smartphone devices used and *N* = *n* + 1 = 5, which includes the reference weather station having a measurement value of *x*_ref_(*t*). Put simply, it is the average of the barometric pressure values of both smartphone devices and weather station at time *t*. Then, we determined *x*_min⁡_(*t*) and *x*_max⁡_(*t*) by comparing pressure readings from among the 4 devices while excluding the weather station since it is a reference. After comparing the smartphone-based pressure readings, we determine the highest pressure value as *x*_max⁡_(*t*) and the lowest as *x*_min⁡_(*t*). In general, determining *α* can give us a quick and general idea on how much the pressure readings in the smartphone differ with the Vaisala WXT520. Moreover, it is also helpful in knowing how close are the pressure readings among different device models. A sample calculation result can be found at Table 2 based on the sample data in Table 3 where the average uncertainty of smartphone-based sensors for 10 minutes of observed barometric pressure was 2.13. Therefore, in our actual measurements of a stationary user with the devices in the shoulder bag, we can express that the pressure may be approximately ±2 precise with Vaisala in reference to the sample calculations.

**Figure 2:**
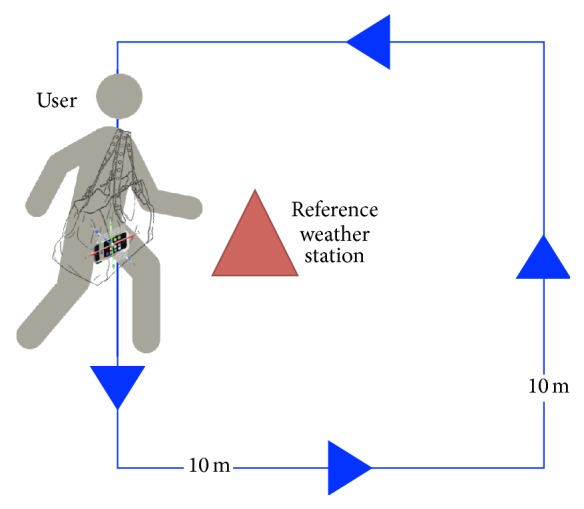
Setup of the moving user experiment.

**Figure 3:**
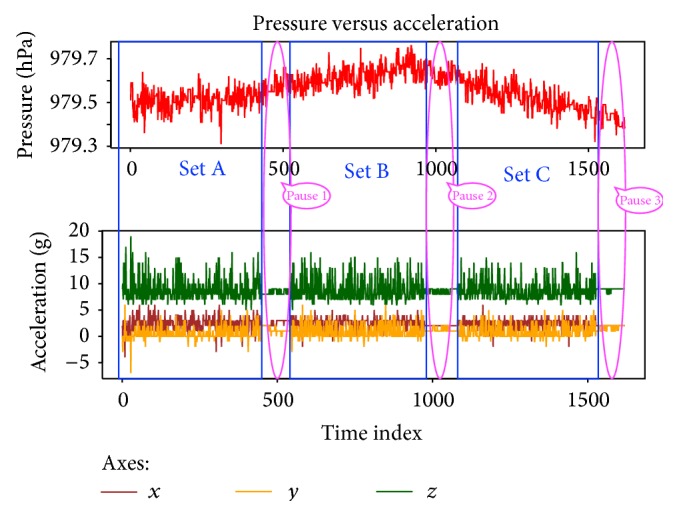
Division of moving and stationary partitions each at 7 and 2 minutes, respectively.

The second experiment, as in Figure 2, was performed to observe the effects of user motion on the pressure readings on the smartphone. As a basic scenario for our proposed system, we imagine a user passing by a reference sensor, which is the kind of user motion that we would like to investigate with this experiment. With the same setup, the user at this time was asked to move around the weather station by walking in a leisurely manner. Each set was composed of 10 rounds, to obtain sufficient sample, that was about 7 minutes long while pausing for 2 minutes in between sets. The movement pattern was designed to estimate the duration of the rounds so we can replicate the same duration, which was 7 minutes for each round. The pause was done to compare moving and stationary events and serve as a marker between sets.  Figure 3 illustrates the raw readings of pressure and accelerometer data taken from the Samsung S4 device as an example. In the chart, the stages of walking and pauses can be easily distinguished by the instability and stability of the accelerometer readings, respectively.

To closely examine the difference between pressure measurements during the presence or absence of movement, we first divided the raw pressure data into partitions of the corresponding stable and unstable measurements of acceleration. This division is shown in Figure 3, where we have three sets of user movement which correspond to an unstable acceleration and three sets in which the user is not moving which correspond to a stable acceleration. Then, we calculated the variance for each partition and the results are shown in Table 4.

Examining the variance of pressure measured between movement and inactivity can indicate the ability of the sensor to stabilize its readings even when subjected to physical disturbance. Weather stations generally follow a standard for fixed setups to provide accurate and precise readings uniformly without having to consider the effect of movement. However, as we are dealing with portable sensors, this is one aspect that we need to further consider for producing reliable measurements similar to that of fixed weather stations. We hypothesize then that the variance of the sensor is higher during movement than when the user is inactive, and thus, we can presume that the sensor is unstable and stable, respectively. To verify this, an upper one-tailed *F*-test was performed between phases of walking and inactivity as shown in the results of Table 5. The results show that the *F* value for all comparisons is greater than the *F*_critical_ values, which rejects the null hypothesis that the variances are equal and proves that the variance of pressure values during movement is higher than when the user is inactive.

Overall, we found that the smartphone pressure sensor reading has an uncertainty value of ±2 when compared with Vaisala WXT520 from our first experiment. Moreover, we verified via an upper one-tailed *F*-test that the variance of the smartphone-based pressure readings is higher during user movement than when the user is stationary. Although the pressure readings were not explicitly proven to be accurate in the experiments, this would still imply that the embedded sensor is more stable in providing pressure readings if the user handling the device is stationary as opposed to when the user is moving.

## 4. Smartphone-Based Sensor Calibration via Pairwise Gossip

Based on our findings on the effect of motion on smartphone sensor readings of pressure, we present our heuristic-based pairwise gossip algorithm. To calibrate embedded smartphone sensors with respect to a fixed weather station, our algorithm relies on the variance of the pressure readings. It is also based on the actual difference of the pressure readings between the smartphone-based sensor and the fixed weather station. Gossip algorithms [34] are generally used for the classic estimation of values in a network by distributed averaging. This particular algorithm has its advantages for distributed averaging in sensor networks as it enables quick and efficient analysis of distributed data over sensor networks especially when faced with several constraints as emphasized in [35]. Such constraints include the lack of centralization, dynamically changing network topology, and sensor hardware limitations. The standard gossip algorithm is in the following form:

(2) $$\begin{array}{c} x\left(t+1\right)=W\left(t\right)x\left(t\right), \end{array}$$

where *W*(*t*) is random weight matrix and *x*(*t*) is the current value of a node in a network. Ideally for gossip algorithms, the weights must converge to a value of 1. In actual pairwise gossiping as stated in [34], random pairs of neighboring nodes exchange their information and calculate the average of their values as some time *t* and update their values with the average, thereafter. In this paper, we apply the same principle of pairwise gossiping by maintaining a one-to-one pairing with the weather station to calibrate the embedded smartphone sensors. However, instead of a random weight assignment, we calculate the weights that equate to a unit value based on the variance of the smartphone-based measurement and the actual difference of the measurements between the smartphone-based sensors and reference weather station.

Let us first consider the following sensing model equation [34]:

(3) $$\begin{array}{c} z_{i}\left(t\right)=H_{i}\theta +w_{i}\left(t\right), \end{array}$$

where *θ* is the value that we want to estimate with our actual pressure readings in the smartphone. In our case, we assign it as our reference value which is the weather station measurement. Meanwhile, *H*_*i*_ and *w*_*i*_(*t*) are the gain and offset of the system in place, respectively. Ideally, *H*_*i*_ = 1 and *w*_*i*_(*t*) = 0 are true if, for instance, the embedded pressure sensor in the smartphones behaves similarly to Vaisala. However, in reality, we have the effects of the surrounding environment, user activity, sensor limitations, and so forth. To explain this concept further, we formulate the following heuristics-based pairwise gossip algorithm to adjust and update the pressure readings in the smartphone as in

(4) $$\begin{array}{c} x_{i}\left(t+1\right)=W_{\alpha }\theta_{i}\left(t\right)+W_{\beta }x_{i}\left(t\right), \end{array}$$

where *x*_*i*_(*t* + 1) is our updated pressure reading (*z*_*i*_(*t*) or *y*). Meanwhile, *W*_*α*_ *θ*_*i*(*t*)_ (*H*_*i*_ *θ* or *mx*) is our reference value *θ* for some ratio of *W*_*α*_. Finally, *W*_*β*_ *x*_*i*_(*t*) (*w*_*i*_(*t*) or *b*) is some ratio of *W*_*β*_ based on variance (Var(*t*)) of *x*_*i*_(*t*) and actual difference (AbsDiff(*t*)) of *x*_*i*_(*t*) from *θ*. The current heuristics algorithm is applicable to a one-to-one calibration of smartphone-based data with a reference weather station. To use the algorithm, the scenario requires that the smartphone is measuring within coverage area of the weather station and consequently located adjacent to the weather station. Thus, (4) presently does not take distance into consideration in the calculation. Furthermore, it follows that *W*_*α*_ + *W*_*β*_ = 1 considering that the weights ideally converge to one. And as for our heuristics-based pairwise algorithm, since we only need to compare two values every time, we simply assigned *W*_*α*_ = 1 − *W*_*β*_, where *W*_*β*_ is the ratio of Var(*t*)/AbsDiff(*t*). To further understand the process of obtaining *W*_*α*_ and *W*_*β*_, we will use a dataset of raw barometric pressure logged by all devices used from one of our measured events as in Table 6.

Referring to S3(1)_raw_ as a more specific example for our calculation process, we first determine the median per minute of the raw pressure readings S3(1)_raw_. This will produce $\text{S}3{\left(1\right)}_{\tilde{x}}$ values that are in the similar temporal resolution as the weather station measurements of pressure. Refer to Table 3 for a sample result of these median values per minute for each device. Then, we calculate the variance of the pressure readings of the raw data of each device per minute or Var_S3(1)_raw__(*t*), for example. Next, we obtain the absolute difference of pressure readings between the calculated medians per minute, ${\text{AbsDiff}}_{\text{Vaisala}-\text{S}3{\left(1\right)}_{\tilde{x}}}(t)$, for instance, at each device and of the weather station measurements. Refer to Table 7 for the results of Samsung S3 as an example of these calculations.

Then, we consider the effects of the user motion via the variance of the smartphone-based readings and the actual difference of the readings between the smartphone-based sensor and the weather station. We do this by calculating the ratio between Var_(S3(1))_raw__(*t*) and AbsDiff_Vaisala−S3(1)_median__(*t*), which we refer to as our *W*_*β*_. Then, we obtain *W*_*α*_ by 1 − *W*_*β*_ considering the prior weight condition that requires the weights equal to one. Finally, using these calculated weights, we can update the value of *x*_*i*_(*t* + 1) as in Table 8.

**Figure 4:**
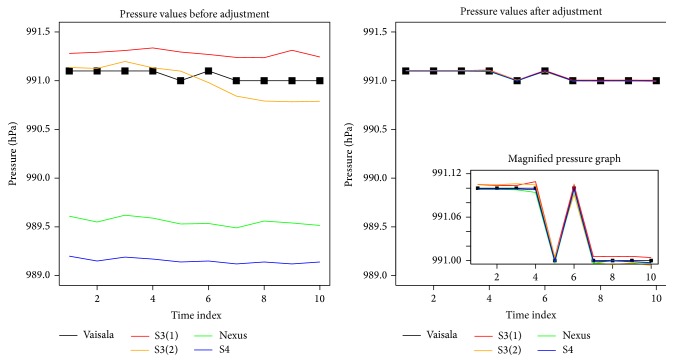
Comparison of pressure per minute before and after adjustments.

The resulting adjustments have significantly transformed the measurements and those measurements are now very close to the reference values as shown in the comparison graph in Figure 4. Each device model essentially has different values of *W*_*α*_ and *W*_*β*_ as reflected in some sample values in Tables 9 and 10, respectively.

In summary, we formulated a heuristic-based pairwise gossip algorithm that adjusts the smartphone measurement values with respect to the weather station measurement. Prior to this, we verified that the variance is higher, for instance, when the user is moving as opposed to when it is stationary. The difference in variance can be linked to the stability and instability of the embedded smartphone sensors. Therefore, to employ this finding, we calculated the weights in accordance with the ratio of the variance of the raw pressure data and the actual difference between the median pressure data and the reference weather station values. These weight calculations apply to calibrating embedded smartphone sensors with fixed weather stations as an established ground truth. Moreover, the weights *W*_*α*_ and *W*_*β*_ are not constant over time. In real measurements, therefore, we can calibrate smartphone-based measurements based on the weights even when the user is moving. For instance, in a setup where the user is located within the coverage of a fixed weather station, the established ground truth measurements would most likely have a larger percentage in the calibration. If the ground truth measurements are presumed to be accurate, these values can be representative estimates of the synoptic ground weather condition. Thus, the percentage of the supporting weather information from the smartphone sensor data is dependent on the weights whereby the effect of movement is mitigated via the variance and absolute difference. As a result of a one-to-one fixed setup, the smartphone sensors would be updated with values closer to the representative estimate.

## 5. Conclusion and Future Work

In this paper, we showed a heuristic-based pairwise gossip algorithm to adjust embedded smartphone pressure sensor measurements. Based on our experiments with the smartphone pressure sensors, we found that the pressure sensors of the different Samsung smartphone models we used have a certain precision value compared with Vaisala WXT520 which we established as our referential ground truth. Moreover, the pressure readings were verified to be unstable when the user is moving compared to when the user is stationary. Thus, to adjust accordingly, we consider the effect of user activity while the device is measuring from inside a shoulder bag by integrating the variance of the raw pressure readings with respect to the actual difference from the reference weather station as our weight ratio. These weight ratios are then consolidated with the pairwise gossip algorithm which updates the pressure reading of the embedded smartphone sensor.

By adjusting the sensor measurements accordingly, we can provide an almost accurate and precise synoptic weather information to general forecasting services. Moreover, as this information can possibly be densely available due to the popular use of smartphones, general weather forecasting services can mitigate errors at the sensor level with this particular calibration method. Thus, this paper contributes a straightforward and heuristic linear estimation using the principles of pairwise gossip. A limitation of this method, however, is that the smartphone requires to be located nearby a weather station at present.

As future work, we plan to extend and update our algorithm to consider scalable and real-time smartphone-based measurements. Consequently, the distance of the mobile device from the reference weather station and from other nearby devices has to be considered and included in the calibration algorithm. Moreover, the method will be improved such that calibration is possible even without a reference weather station. For one device calibrated nearby a reference weather station, all other neighboring smartphone devices can be calibrated in reference to it. The smartphone can then retain the calibration information and may not constantly need a reference weather station. Also, we may have to consider other environmental sensors like the temperature and humidity and determine if the same algorithm and principles apply.

## Figures and Tables

**Table 1:** List of smartphone models used and their available sensors.

Common name	Galaxy Nexus [16]	S3 [17]	S4 [18]
Model	I9250	GT-I9300	GT-I9500

Android OS version	4.3	4.2.2	4.3

Light (lux)	✓	✓	✓
Proximity (cm)	✓	✓	✓
Gyroscope (rad/s)	✓	✓	✓
Accelerometer (m/s^2^)	✓	✓	✓
Magnetometer (*μ*T)	✓	✓	✓
Pressure (hPa)	✓	✓	✓
Temperature (°C)	N/A	N/A	✓
Humidity (%)	N/A	N/A	✓

**Table 2:** Uncertainty calculation results based on the Vaisala WXT520.

Time	$\bar{X(t)}$	*x*_min⁡_(*t*)	*x*_max⁡_(*t*)	Uncertainty
14:00	990.47	989.20	991.28	2.08
14:01	990.44	989.15	991.29	2.14
14:02	990.48	989.19	991.31	2.12
14:03	990.47	989.17	991.34	2.167
14:04	990.41	989.14	991.29	2.15
14:05	990.41	989.15	991.27	2.12
14:06	990.34	989.12	991.24	2.12
14:07	990.35	989.14	991.24	2.10
14:08	990.35	989.12	991.31	2.19
14:09	990.34	989.14	991.24	2.10

**Table 3:** Sample pressure data for uncertainty calculation.

Time	Vaisala	$\text{S}3{\left(1\right)}_{\tilde{x}}$	$\text{S}3{\left(2\right)}_{\tilde{x}}$	$\text{Nexu}{\text{s}}_{\tilde{x}}$	$\text{S}4_{\tilde{x}}$
14:00	991.1	991.28	991.14	989.61	989.20
14:01	991.1	991.29	991.13	989.55	989.15
14:02	991.1	991.31	991.2	989.62	989.19
14:03	991.1	991.34	991.13	989.59	989.17
14:04	991	991.29	991.1	989.53	989.14
14:05	991.1	991.27	990.98	989.54	989.15
14:06	991	991.24	990.84	989.49	989.12
14:07	991	991.24	990.79	989.56	989.14
14:08	991	991.31	990.78	989.54	989.12
14:09	991	991.24	990.79	989.51	989.14

**Table 4:** Sample result of calculated variance between partitions of moving and stationary user.

Partition name	Variance
Set A	0.0021
Pause 1	0.0018
Set B	0.0025
Pause 2	0.0013
Set C	0.0032
Pause 3	0.0017

**Table 5:** *F*-test result for a sample observation.

Partition	*F*	*F*_critical_
Set A versus Pause 1	1.18	0.92
Set B versus Pause 2	1.91	1.49
Set C versus Pause 3	1.82	1.45

**Table 6:** Sample raw data from a 10-minute event.

Time	S3(1)	S3(2)	Nexus	S4
14:00:00	991.39	991.11	989.57	989.16
14:00:01	991.35	991.14	989.6	989.16
14:00:02	991.33	991.16	989.58	989.16
⋮	⋮	⋮	⋮	⋮
14:09:59	991.23	990.90	989.58	989.14

**Table 7:** S3(1) calculation result of variance and absolute difference.

Time	Vaisala	$\text{S}3{\left(1\right)}_{\tilde{x}}$	Var(*t*)	AbsDiff(*t*)
14:00	991.1	991.28	0.0049859	0.17978
14:01	991.1	991.29	0.0037714	0.19140
14:02	991.1	991.31	0.0037007	0.20952
14:03	991.1	991.34	0.0090520	0.23600
14:04	991	991.29	0.0046557	0.29380
14:05	991.1	991.27	0.0047261	0.16914
14:06	991	991.24	0.0055904	0.23865
14:07	991	991.24	0.0054008	0.23590
14:08	991	991.31	0.0056691	0.31200
14:09	991	991.24	0.0041641	0.24316

**Table 8:** S3(1) calculation result of *W*(*t*) and *x*_*i*_(*t* + 1).

Time	Vaisala	S3(1)	*W*_*α*_	*W*_*β*_ = 1 − *W*_*α*_	*x*_*i*_(*t* + 1)
14:00	991.1	991.28	0.027733	0.97227	991.1
14:01	991.1	991.29	0.019704	0.98030	991.1
14:02	991.1	991.31	0.017663	0.98234	991.1
14:03	991.1	991.34	0.038356	0.96164	991.11
14:04	991	991.29	0.015847	0.98415	991
14:05	991.1	991.27	0.027943	0.97206	991.1
14:06	991	991.24	0.023425	0.97657	991.01
14:07	991	991.24	0.022894	0.97711	991.01
14:08	991	991.31	0.018170	0.98183	991.01
14:09	991	991.24	0.017125	0.98287	991

**Table 9:** Calculated *W*_*α*_ of different device models.

Time	S3(1)	S3(2)	Nexus	S4
14:00	0.97227	0.84940	0.99894	0.99937
14:01	0.98030	0.81662	0.99896	0.99942
14:02	0.98234	0.93642	0.99852	0.99945
14:03	0.96164	0.85621	0.99615	0.99890
14:04	0.98415	0.95145	0.99813	0.99932
14:05	0.97206	0.92999	0.99847	0.99957
14:06	0.97657	0.97966	0.99752	0.99920
14:07	0.97711	0.97323	0.99998	0.99971
14:08	0.98183	0.98382	0.99859	0.99926
14:09	0.98287	0.96791	0.99768	0.99845

**Table 10:** Calculated *W*_*β*_ of different device models.

Time	S3(1)	S3(2)	Nexus	S4
14:00	0.027733	0.15060	0.0010565	0.00062344
14:01	0.019704	0.18338	0.0010387	0.00057949
14:02	0.017663	0.063578	0.0014766	0.00055123
14:03	0.038356	0.14379	0.0038511	0.0010974
14:04	0.015847	0.048554	0.0018666	0.00067643
14:05	0.027943	0.070009	0.0015258	0.00043013
14:06	0.023425	0.020340	0.0024820	0.00079598
14:07	0.022894	0.026771	0.000017361	0.00029228
14:08	0.018170	0.016183	0.0014091	0.00074171
14:09	0.017125	0.032091	0.0023210	0.0015459
